# The Bidirectional Signal Communication of Microbiota-Gut-Brain Axis in Hypertension

**DOI:** 10.1155/2021/8174789

**Published:** 2021-12-21

**Authors:** Xiaoqi Wang, Zhenzhen Chen, Bin Geng, Jun Cai

**Affiliations:** FuWai Hospital, State Key Laboratory of Cardiovascular Disease, National Center for Cardiovascular Diseases, Peking Union Medical College, Chinese Academy of Medical Sciences, Beilishi Rd. 167, Xicheng District, Beijing 100037, China

## Abstract

Hypertension is a critical risk factor of cardiovascular diseases. A new concept of microbiota-gut-brain axis has been established recently, mediating the bidirectional communication between the gut and its microbiome and the brain. Alterations in bidirectional interactions are believed to be involved in the blood pressure regulation. Neuroinflammation and increased sympathetic outflow act as the descending innervation signals from the brain. Increased sympathetic activation plays a recognized role in the genesis of hypertension. The present evidence demonstrates that gut dysbiosis is associated with central nervous system neuroinflammation. However, how the gut influences the brain remains unclear. We reviewed the roles of neuroinflammation and gut microbiota and their interactions in the pathogenesis of hypertension and described the ascending signaling mechanisms behind the microbiota-gut-brain axis in detail. Additionally, the innovative prohypertensive mechanisms of dietary salt through the microbiota-gut-brain axis are summarized. The bidirectional communication mechanisms were proposed for the first time that the descending signals from the brain and the ascending connections from the gut form a vicious circle of hypertension progression, acting as a premise for hypertension therapy.

## 1. Introduction

Researchers estimated mortality from cardiovascular diseases, chronic kidney disease, and diabetes all over the world from 1980 to 2010, and high blood pressure (BP) was the leading risk factor for deaths due to these diseases throughout the analysis period [1]. Therefore, innovative approaches to effectively prevent and manage hypertension are urgently needed. Microbiota-gut-brain axis refers to a bidirectional communication between the gut microbiota (GM) and the brain [2]. Alterations in this bidirectional interactions are believed to be involved in BP regulation, which may provide a new way to treat hypertension in the near future [3].

Cardiovascular brain centers regulate BP by controlling sympathetic and parasympathetic activities. As the sympathetic nervous system innervates multiple organs, it controls key pathophysiological processes in the BP regulation such as vasoconstriction, water-sodium balance, and renin-angiotensin system (RAS) activity and regulates systemic inflammation status by innervating the gut and bone marrow [4]. Neuroinflammation of the central nervous system (CNS) leads to increased sympathetic activation. Recent study highlighted that the alterations of GM composition associated with the neuroinflammation and sympathetic nervous system to mediate BP regulation [5]. The evidence suggests a potential linking axis between the GM and neuroinflammation in the CNS.

Recent basic and clinical findings indicate that gut dysbiosis is a novel causation of hypertension initiation and development. The evidences include (1) reciprocal interactions between the GM and cardiovascular disease risk factors (such as obesity, insulin resistance, and chronic inflammation) [6]; (2) the remarkable alterations of the GM in hypertensive cohorts and experimental models [7–10]; (3) fecal transplantation evidence [8, 11]; (4) antihypertensive properties of intervention strategies to correct gut dysbiosis.

An increased Firmicutes/Bacteroidetes ratio and a significant decrease in microbial richness, diversity, evenness, and short-chain fatty acids (SCFAs)-producing bacteria in the spontaneously hypertensive rats (SHRs) have been described [12]. Additionally, another study has showed taxonomic and functional changes in the gut microbiome, especially the significant reduction in butyrate-producing bacteria, aberrant gut barrier function, and increased local inflammation in hypertensive patients [13]. Compared to healthy controls, both prehypertensive and hypertensive populations show decreased microbial richness and diversity, distinct metagenomic composition with reduced bacteria associated with healthy status and overgrowth of harmful bacteria, and disease-linked microbial functions [8]. As an efficient method to demonstrate the causal role of the GM in hypertension, fecal transplantation can regulate BP in different animal models [7, 9]. Hypertension could be induced in a normotensive strain (normotensive Wistar–Kyoto (WKY), normotensive OSA) of rats or attenuated in a hypertensive strain [SHRs, hypertensive OSA] of rats by exchanging the GM between the two strains [7, 9]. Also, by fecal transplantation from hypertensive human donors to germ-free mice, high BP was observed to be transferrable through the GM [8].

Salt, as one of the most common prohypertensive factors, added or inherent to food contributes to nearly 99% of the total sodium intake [14]; thus, the effects of dietary salt on the GM and brain need to be elucidated. Recent studies demonstrated that excess salt intake exerts a certain effect on the composition of the GM [15]. Also, dietary salt is approved to cause neuroinflammation and sensitize central sympathetic circuits [14, 16]. However, the role of salt intake on the microbiota-gut-brain axis remains unclear.

The aim of this review is to provide a brief summary of the current knowledge with a focus on neuroinflammation, GM, and their interactions in the pathogenesis of hypertension, as well as the specific signaling mechanisms behind the microbiota-gut-brain axis, and to provide an innovative insight into prohypertensive mechanisms of dietary salt through the microbiota-gut-brain axis.

## 2. Neuroinflammation in Hypertension

**Figure 1:**
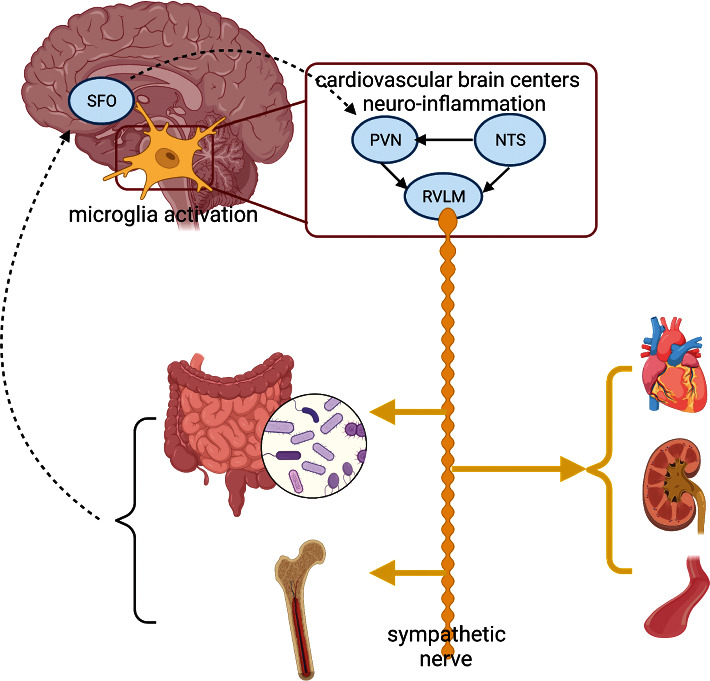
The hypothetic mechanisms of neuroinflammation-mediated bidirectional communication in the microbiota-gut-brain axis.

The neuroinflammation in cardiovascular brain centers leads to the imbalance of sympathetic/parasympathetic activity, and increased sympathetic activation plays a recognized role in the genesis of hypertension. Increased sympathetic activation affects the target organs which regulate BP, such as the blood vessels, kidney, and heart [4], and also leads to the peripheral and central immune system inflammatory activation by directly stimulating bone marrow [17]. In addition, elevated sympathetic activity in the gut is able to change the components of the GM, contributing to the low-grade inflammation associated with hypertension [18] (Figure 1).

The schematic illustration shows that activated microglia regulate the autonomic nuclei circuits (PVN, NTS, and RVLM) in cardiovascular brain centers and cause neuroinflammation in these brain regions. The neuroinflammation in cardiovascular brain centers leads to the overdrive of the sympathetic system. The sympathetic nervous system innervates multiple organs (peripheral vasculature, kidney, bone marrow, and gut) and controls key pathophysiological process in the onset and progression of hypertension. Gut dysbiosis and central immune system activation in bone marrow associated with the overdrive of the sympathetic system lead to systemic inflammation. The SFO can sense peripheral pro-inflammatory cytokines, project directly to the PVN, and activate microglia, bridging the systemic inflammation and neuroinflammation. Abbreviations are as follows: hypothalamic paraventricular nucleus (PVN), rostral ventrolateral medulla (RVLM), nucleus tractus solitaries (NTS), and subfornical organ (SFO).

### 2.1. Neuroinflammation in Cardiovascular Brain Centers

Cardiovascular brain centers are located in the hypothalamus and brainstem, including several important nuclei such as the hypothalamic paraventricular nucleus (PVN), the rostral ventrolateral medulla, and the nucleus tractus solitaries (NTS). PVN is the predominant autonomic region that accumulates activated microglia exhibiting neuroinflammation in hypertension [19]. These nuclei influence BP by regulating the activity of the autonomic nervous system [20].

The observation that a variety of pro-inflammatory cytokines are upregulated in the cardiovascular brain region of hypertensive animals underscores the link between CNS neuroinflammation and hypertension [18]. Administration of pro-inflammatory factors such as tumor necrosis factor-(TNF)-*α* or interleukin-(IL)-1*β* into cardiovascular brain centers can increase sympathetic tone, brain RAS activity, and BP, while central down-regulation of these factors has a significant opposite effect [21]. To explore the source of central pro-inflammatory cytokines and the specific cause of neuroinflammation in hypertension, it is important to emphasize the role of activated resident microglia.

### 2.2. Activated Microglia in CNS Neuroinflammation

Microglia are the only resident immune cells in the CNS and are known to participate in both innate and adaptive immune responses to infection and injury. However, activation of microglia can be a two-edged sword. Activated microglia produce many pro-inflammatory mediators—including cytokines, chemokines, reactive oxygen species, and nitric oxide—which contribute to the clearance of pathogen infections, but prolonged or excessive activation may result in pathological neuroinflammation [22]. Activated microglia show a universe of activation states, and M1 (“classical” activation) and M2 (“alternative” activation) represent extremes of this continuum [22].

Microglia can become activated and/or dysregulated in the context of hypertension and can exacerbate hypertension through augmenting neuronal excitation, such as the overdrive of the sympathetic neuron [23, 24]. Ang-II is one of the signals that can activate resident microglia in cardiovascular brain centers. In addition, many other prohypertensive factors have also been proved to be able to activate microglia [25]. This may partially illustrate the reason why microglia is activated in hypertension. Also, gut-derived lipopolysaccharide (LPS) acting as pathogen-associated molecular patterns (PAMPs) can be recognized by pattern recognition receptors (PRRs) on the microglia membrane and activate the local immune response.

Though M1 microglia are thought to be associated with chronic inflammation disease states, the specific activation form of microglia in hypertension has not been determined. Using deoxycorticosterone acetate (DOCA)-salt-treated rat model mimicking sporadic and chronic hypertension, Koizumi et al. showed that microglia juxtaposed to the vessels directly switched to the pro-inflammatory M1 state after 3 weeks, differing from M2-to-M1 class switching in acute ischemic models [24]. However, in the model induced by either angiotensin II (Ang-II) or L-NG-nitro-l-arginine methyl ester (L-NAME), both M1 and M2 markers were upregulated [26]. The interactions among these signaling pathways and the specific molecule mechanisms mediating microglia activation in hypertension remain to be elucidated.

### 2.3. Microglial Activation Exacerbates Hypertension

Microglial activation in established hypertension is detrimental to neural homeostasis and exacerbates the disease [23]. Targeted depletion of microglia in PVN attenuated neuroinflammation and high BP caused by either Ang-II or L-NAME. By contrast, adoptive transfer of preactivated microglia into the brains of normotensive mice prolonged pressor responses [26]. Hypertension leads to cerebrovascular histopathological alterations including structural changes such as hypertrophy, remodeling, stiffening, and vascular regulation insufficiency [27]. Studies have proved that microglial activation preceded the appearance of histopathological abnormalities, thereby underscoring the possible role of microglia in the process of hypertension-induced cerebral vessel damage [24]. Considering these findings, we can conclude that microglial activation is one of the hallmarks and crucial regulating factors in the process of hypertension. As for what causes the activation of microglia, the gut is one of the key factors that cannot be ignored.

## 3. Signaling Mechanisms behind the Microbiota-Gut-Brain Axis

**Figure 2:**
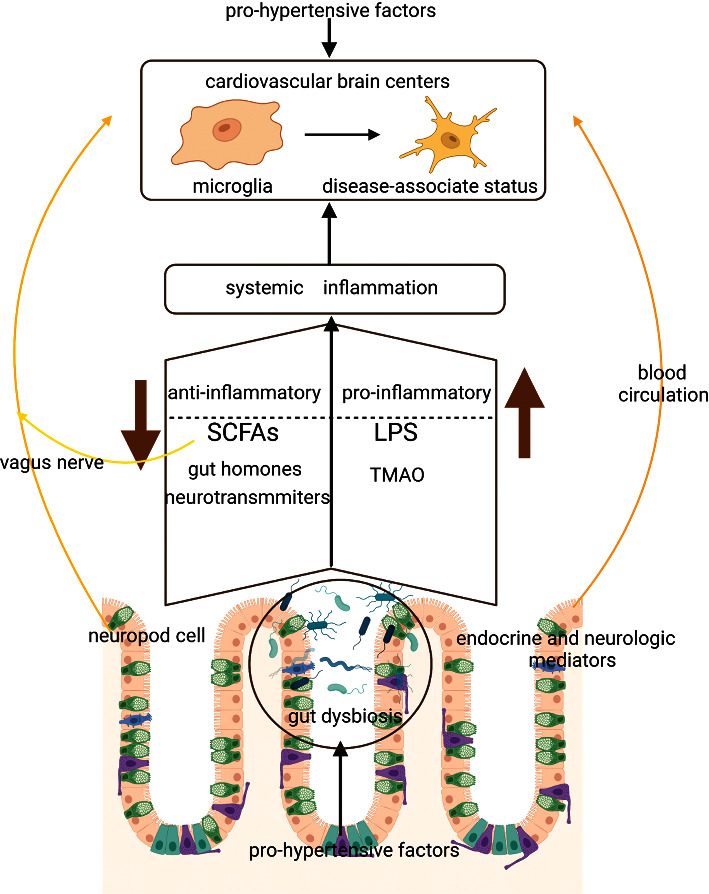
Ascending connections through the immune system and the vagus nerve in the microbiota-gut-brain axis.

To highlight the complex communication between the gut, its microbiome, and the brain, the concept of the microbiota-gut-brain axis has been proposed [2]. Current evidence suggests that multiple mechanisms may be involved in GM-to-brain signaling and that the brain can in turn alter the GM via the autonomic nervous system [28]. Changes in these bidirectional interactions are believed to be involved in the pathogenesis of hypertension though the evidence is still limited [3]. In the following context, we proposed that enhanced sympathetic tone acts as the signal from the brain innervating multiple target organs of BP regulation, and both the immune system and the vagus nerve contribute to the ascending connections in the microbiota-gut-brain axis (Figure 2).

The multiple routes of ascending connections between the gut microbiota and the brain are being revealed. Gut dysbiosis in the context of hypertension leads to the imbalance of circulatory anti-and pro-inflammatory mediators and host systemic inflammation. Systemic inflammation can directly or indirectly activate microglia and induce neuroinflammation. The colonic afferent vagus nerve projects to the cardiovascular brain center and is able to transmit a variety of gut signals to the CNS, modulating the important nuclei and their circuits involved in the control of autonomic nervous system activity. Abbreviations are as follows: LPS: lipopolysaccharides, TMAO: trimethylamine-N-oxide, and SCFAs: short-chain fatty acids.

### 3.1. Gut Microbiota and Neuroinflammation

A human individual carries up to 100 trillion resident bacteria, outnumbering the body cells tenfold [29]. The gut is one of the largest endocrine, immune, and neural organs in the body, constituting a huge substantial microbial habitat, and the gut flora has a metabolic activity equal to a virtual organ within an organ [30, 31]. Accumulating evidence has revealed an important role of the GM in the development of hypertension [32].

The GM can establish efficient crosstalk with the rest of the body and influence the host inflammatory status through a number of mediators because a significant part of the metabolites in circulation are derived from the GM [33]. There are experimental data to show that changes of the GM composition are able to influence the neuroinflammation in cardiovascular brain centers, acting as the ascending connection in the microbiota-gut-brain axis [5].

SCFAs are a kind of organic fatty acid with less than six carbon atoms [34]. As the byproducts of bacterial fermentation of nondigestible carbohydrates and resistant starch, the SCFAs acetate, propionate, and butyrate are the most abundant [35, 36]. However, most gut-derived SCFAs are ingested by gut epithelial cells and hepatocytes, with only about 36%, 9%, and 2% of the acetate, propionate, and butyrate, respectively, reaching the systemic circulation to influence target organs and tissues, including the brain [37]. In addition to functioning in the CNS, SCFAs can interact with vagal nerve afferents which project to NTS, arguing for a potential key role of SCFAs in the microbiota-gut-brain axis [36]. Studies have shown that SCFAs are able to decrease BP through the abilities of anti-inflammation and neuroprotection [38]. Given that SCFAs are vital mediators by which the GM regulate the BP, it is reasonable to assume that using SCFAs directly may be a possible intervention of high BP. In the chronic Ang-II infusion hypertensive mouse model, butyrate supplementation could ameliorate high BP, consistent with the hypothesis [13]. Further interventional studies in humans are needed to determine whether there is a cause-and-effect relationship between SCFAs intake and reduced blood pressure.

### 3.2. Ascending Connections through the Immune System

The GM is able to manipulate the inflammatory status of the host through an array of mechanisms. Studies have found that in the context of hypertension: (1) GM-derived proinflammatory mediators increase, including LPS, trimethylamine-N-oxide (TMAO), and sulfate [39–41]; while anti-inflammatory mediators such as SCFAs decrease. The imbalance of anti-inflammatory and proinflammatory mediators in blood circulation leads to host systemic inflammation. (2) The GM can affect the gut epithelium in producing anti-inflammatory gut hormones and neurotransmitters in an indirect way [42]. Compared with the normotensive WKY rats, the SHRs showed a >30% increase in bone marrow and blood inflammatory cells [17]. Under many circumstances, the immune cells and cytokines of peripheral blood circulation can break the “barrier isolation” called the blood-brain barrier (BBB) to act on the brain. LPS plays a recognized role in activating systematic inflammation. In a hypertensive patient cohort, plasma LPS levels and gut-target proinflammatory T helper 17 (Th17) cells were significantly increased [13]. In SHR models, along with the increase in BP, studies observed increased intestinal permeability and decreased tight junction proteins [43]. Furthermore, the hypertensive gut microbiome exhibited higher LPS biosynthesis [10, 44]. Therefore, LPS produced by the GM increases in hypertensive patients as a result of microbiota dysbiosis, and more gut-derived LPS leak into blood circulation due to the increase in intestinal permeability worsen the systemic inflammation.

In humans, the systemic inflammation induced by intravenous injection of LPS activates microglia in the brain and leads to neuroinflammation [45]. Systemic LPS increase results in CNS neuroinflammation mainly through two pathways. As the BBB dysfunction can be found in the early stage or even before the onset of hypertension, systemic LPS can directly cross the aberrant BBB [46]. Microglia express PRRs that recognize various PAMPs including gut-derived LPS [22]. Following the recognition of PAMPs by microglia, PRR-mediated signal transduction induces the classical M1-like activation and innate immune response.

Also, gut-derived LPS can enter the blood circulation and binds to toll-like receptor 4 on innate immune cells, leading to the increase in peripheral proinflammatory cytokines. As a unique brain region that lacks a BBB, the subfornical organ (SFO) is an important brain sensor of peripheral proinflammatory cytokines, mediating their central effects on cardiovascular brain centers [47]. Microinjection of TNF-*α* or IL-1*β* (mimicking systemically administered proinflammatory cytokines) into the SFO elevated the BP and sympathetic outflow [47]. The SFO projects directly to the PVN, and activates the microglia in the PVN [48]. This is likely why systemic inflammation can influence autonomic brain regions and cardiovascular function.

Along with LPS, trimethylamine-N-oxide (TMAO) is another proinflammatory mediator generated by the GM. Increased circulating levels of TMAO directly activates inflammatory pathways in cells of the vasculature, leading to endothelial cell leukocyte recruitment [39].

SCFAs are able to decrease BP through the abilities of anti-inflammation and neuroprotection [38]. Acetate, propionate, and butyrate are detectable in the human cerebrospinal fluid at physiological concentrations [35]. Studies have shown that sodium butyrate mediates neuroprotection though modifying microglial activation modes [38]. Likewise, the acetate treatment of microglia in primary culture has been shown to reduce inflammatory signaling through downregulation of IL-1*β* and TNF-*α* expression and p38 MAPK, JNK, and NF-*κ*B phosphorylation [49]. Gut-derived SCFAs exert an influence on hosts based on two major pathways: (1) SCFAs enter the target cells mediated by transporters (MCTs and SMCTs) and directly inhibit histone deacetylases (HDACs), consequently regulating the downstream gene expression, and (2) SCFAs bind to G protein-coupled receptors to activate related cellular signal transduction pathways [50–55]. Without G protein-coupled receptor corresponding to SCFAs on the microglial membrane, gut-derived SCFAs influence microglia based on HDAC inhibition. Gut-derived SCFAs acting as HDAC inhibitors exhibit immunosuppressive effects by epigenetically regulating the microglial inflammatory response [38]. Furthermore, reduced circulating butyrate levels caused by impaired transport colonic absorption and reduced responsiveness of the hypothalamic PVN have been found in SHRs [56]. Another research showed that reduction in butyrate-producing bacteria may lead to gut-barrier dysfunction and increase in systemic LPS [13]. SCFAs-producing species can exert the effects of anti-inflammation and neuroprotection by stimulating enteroendocrine cells to produce anti-inflammatory gut hormones, such as glucagon-like peptide 1 (GLP-1), glucagon-like peptide 2 (GLP-2), and peptide YY (PYY), while some species can directly produce the anti-inflammatory neurotransmitters such as *γ*-aminobutyric acid (GABA) and acetylcholine (Ach) [33, 40].

Gut dysbiosis in the context of hypertension leads to the imbalance of circulatory anti- and proinflammatory mediators. Reduced anti-inflammatory mediators result in diminished neuroprotection and anti-inflammation ability. Circulatory LPS increases the level of neuroinflammation through the activation of systemic inflammation and direct recognition of PAMPs by microglia. Thus, the GM levies its effects on cardiovascular brain centers through the immune system, inducing microglial activation and central neuroinflammation.

### 3.3. Ascending Connections through the Vagus Nerve

The colonic afferent vagus nerve projects to the cardiovascular brain center and is able to transmit a variety of gut signals to the CNS, modulating the important nuclei and their circuits involved in the control of autonomic nervous system activity and BP. Specifically, the afferent signal directly projects to the NTS which can modulate the behaviour of the PVN, thus participating in the regulation of sympathetic nerve activity [57]. However, how the afferent vagus nerve can sense the gut signals remains poorly understood.

Electrically excitable sensory cells called the enteroendocrine cells are found dispersed within the gut epithelium, and most enteroendocrine cells communicate indirectly with nerves through hormone secretion but not through a direct synaptic link [58]. Recently, researchers have found a type of gut epithelial cell that forms synapses with vagal neurons and named them the “neuropod cell” [59]. Neuropod cells use glutamate as a neurotransmitter to transduce signals to vagal neurons connecting the gut lumen to the brainstem. Therefore, it can be assumed that specific gut epithelial cells can sense GM signals and transmit them to the cardiovascular brain center by means of nerve and hormone conduction.

In addition, although the vagus nerve cannot contact the GM directly, bacterial metabolites can act on the vagus nerve through the intestinal mucosal barrier. It is recognized that SCFA receptors are expressed in the afferent fibers of the vagus nerve [60]. Administering butyric acid into the colon to increase the concentration by 2-3-fold represents a significant hypotensive effect, and this effect depends on normal function of the afferent colonic vagus nerve and SCFA receptor, GPR41/43 [61]. In summary, SCFAs mediate the communication between the GM and the afferent colonic vagus nerve.

Based on these findings, the ascending connections through the vagus nerve can be transmitted to the brain through the synapses between gut epithelial sensory cells and vagal neurons or through the interaction between SCFAs and the afferent vagus fibers.

## 4. Sodium Salt Intake Influences the Microbiota-Gut-Brain Axis

The intestinal mucosa, loaded with rich immune cells and GM, is the first and main absorption site for excess salt. Diet-induced alterations in the GM have implicated influence on local gut immune systems, especially on T cells [62]. Th17 cells are most abundant at steady state in gut-associated lymphoid tissues, where they accumulate only in the presence of luminal commensal microbiota [63]. High salt intake has been approved to change the GM both in humans and mice, reflecting an increase in Firmicutes, Proteobacteria, and genus *Prevotella* bacteria which were associated with higher BP [15]. More specifically, high salt intake depletes *Lactobacillus murinus* (*L. murinus*) and treatment with *L. murinus* prevented salt-induced aggravation of experimental autoimmune encephalomyelitis and salt-sensitive hypertension by modulating Th17 cells; and also in humans, a moderate high-salt challenge reduced intestinal survival of *Lactobacillus* spp., increased Th17 cells, and increased BP [64]. Another study also linked the excess dietary salt with the GM, inflammation, and hypertension. GM alterations induced by high salt intake are associated with increased costimulatory ligand and IsoLG protein adduct formation in antigen-presenting cells (APCs), which leads to increased intestinal and vascular inflammation and hypertension [65].

Salt is thought to sensitize central sympathetic circuits. Elevations in plasma and cerebrospinal fluid (CSF) Na + enhance sympathetic nerve activity via the RVLM leading to increases in BP [66]. Nonetheless, potential “sensing” mechanisms for Na + existing in the brain have been elucidated using rodent models [67–69]. Central Na + sensing occurs in the circumventricular organs including the organum vasculosum of the lamina terminalis (OVLT) and SFO, which lack an intact BBB [67]. The SFO has been mentioned above as an important brain sensor of peripheral proinflammatory cytokines [47]. This coincidence suggests that both Na+ and inflammatory mediators contribute to neuroinflammation. Like SFO, the OVLT also projects to the PVN, which plays an essential role in regulating sympathetic nerve activation via neuronal projections to the RVLM.

In summary, excess dietary salt can alter the GM and activates gut local and systemic immune systems, and sensitize central sympathetic circuits to elevate BP. The more accurate mechanisms between salt and the microbiota-gut-brain axis need more basic and clinical investigations.

## 5. A Premise for Hypertension Therapy

As the microbiota-gut-brain axis plays an emerging role in hypertension, many studies have explored traditional antihypertensive treatments associated with this crosstalk and GM medication. Despite the breadth of emerging knowledge, current common control and treatment of hypertension are not targeting the overall dysfunctional axis, especially the brain.

Captopril is a classic antihypertensive drug, lowering BP through its suppressive effect on the RAS at both peripheral and central sites. However, recent studies demonstrated that captopril influences the microbiota-gut-brain axis to maintain the sustained antihypertensive effect after withdrawal, exerting significant long lasting influences on GM composition, gut permeability, and pathology as well as brain activity [70]. Similar studies are warranted to validate the translational implications of traditional antihypertensive drugs on the microbiota-gut-brain axis.

Interventions to correct the GM can be seen as an innovative nutritional therapeutic strategy by modulating the microbiota-gut-brain axis to exert the antihypertensive effects, though the effect of decreased CNS inflammation is indirect. Hypertensive OSA rats administered with the probiotic *C. butyricum* or the prebiotic Hylon VII exhibit increased fecal acetate concentration, reduced dysbiosis, and epithelial damage [71]. Blueberries fermented with the tannase-producing bacteria *L. plantarum* DSM 15313 have antihypertensive properties [72]. In elderly people who are initially normotensive, frequent intake of fermented probiotic milk products reduces the risk of developing hypertension [73]. The meta-analysis of nine studies showed that probiotic consumption makes a significant reduction of systolic BP by 3.56 mmHg and diastolic BP by 2.38 mmHg, when compared with the control groups [74]. In the Heart Outcomes Prevention Evaluation (HOPE) study, even modest reduction of systolic BP by 3.3 mm Hg and diastolic BP by 1.4 mmHg led to a significant 22% reduction in relative risk of cardiovascular mortality, myocardial infarction, or stroke [75].

Additionally, lifestyle improvements may be a power approach to ameliorate microbiota-gut-brain axis impairment to induce antihypertensive effects. For example, exercise was associated with improved gut pathology, inflammation, and permeability, enrichment of beneficial bacterial genera, and decreased brain neuroinflammation [76].

## 6. Conclusions

In this review, we focus on the signal communication in microbiota-gut-brain crosstalk associated with hypertension. The descending signal from the CNS is increased sympathetic output. The ascending connections include both the immune system and the vagus nerve. From the existing data, we proposed that hypertensive risk factors (genes, diet, and environment) lead to gut dysbiosis, and this signal transfer from the gut to brain, inducing microglial activation and followed by neuroinflammation. Besides, prohypertensive signals (diet, salt, stress, and Ang-II) perceived in cardiovascular brain centers also activate resident microglia and enhance sympathetic output. The sympathetic nervous system innervates multiple organs and controls key pathophysiological process in the onset and progression of hypertension. The pathological changes of the gut and its microbiome, at least partially caused by increased sympathetic outflow, in turn act on the brain through both immune and neural pathways, forming a vicious circle of hypertension progression. Dietary salt is one of the common prohypertensive factors and influence the microbiota-gut-brain axis in many aspects. Excess dietary salt can alter the GM, activates gut local and systemic immune systems, and sensitize central sympathetic circuits to elevate BP. Limited information is available on how these findings may translate to clinical applications in hypertension involving the microbiota-gut-brain axis, especially targeting the brain neuroinflammation. This new hypothesis is likely to fill fundamental knowledge gaps leading to innovative research, clinical trials, and treatments for hypertension in modulating the microbiota-gut-brain axis.

## Data Availability

All data supporting the conclusions of this review are published papers searched from PubMed. Others can access all the data through the DOIs provided in the references of this review.
